# Defects in, and Suggestions for the Improvement of the Reports of Examining Surgeons1Paper read by invitation at the first annual meeting of the National Association of United States Pension Examining Surgeons, held at Saratoga Springs, N. Y., on June 9, 1902.

**Published:** 1902-10

**Authors:** J. F. Raub

**Affiliations:** Washington, D.C.; Medical Referee, Bureau of Pensions


### BUFFALO MEDICAL JOURNAL.

VOL. XLII.—LVIII. OCTOBER, 1902. NO. 3.

### ORIGINAL COMMUNICATIONS.

### Defects In, and Suggestions for the Improvement of the Reports of Examining Surgeons.[1]

By J. F. RAUB, M. D., Washington, D.C.
Medical Referee, Bureau of Pensions.

NEARLY five years ago, while discussing the duties and requirements of examining surgeons with several of these surgeons who were visiting the bureau, I informed them that I was very much interested in divorcing these agents of the government from the idea that those who were ordered before them had certain peculiar claims upon them. I tried to point out to them that they must not be influenced by the importunities of claimants or their attorneys. I tried to impress upon them the fact that a full performance of their duty consisted in making a full and thorough examination, and furnishing a complete, comprehensive pen picture of the condition found, with even justice to the government and the soldier—that they must always be impartial.

I suggested to them to organise into an association, state or national. I expressed my conviction that such an organisation would result in inestimable good to all, because the meetings would give opportunity to confer about methods, and to discuss plans for improving the service. I was assured then, and have been since, that the suggestion was a good one, and that efforts would be made to secure such an organisation. Nothing came of it, however, until you gentlemen took hold of the matter.

I greet you as pioneers. I congratulate you on your success. I congratulate you on bringing face to face so many of the more

1. Paper read by invitation at the first annual meeting of the National Association of United States Pension Examining Surgeons, held at Saratoga Springs, N. Y., on June 9, 1902.

than 4,500 examining surgeons upon whose faithful, unbiased reports both the government and the great body of Union ex-soldiers necessarily rely for a just adjudication of pension claims. The magnitude of your work and the great interests involved are made manifest by the fact that during the fiscal year ending June 30, 1901, nearly 200,000 examinations were made, as many certificates sent to the bureau, and $910,000 in fees paid therefor.

An experience of eighteen years as an examining surgeon for the bureau gave the present medical referee an appreciation of the labors and responsibilities of the office. His services as a medical examiner and reviewer in the medical division of the bureau for nearly seven years immediately preceding his appointment as medical referee, gave him a very thorough knowledge of the character of the reports, the certificates furnished by examining surgeons.

It was an undisputed fact, admitted by everyone who had to do with the adjudication of claims that, in those days, these reports were, as a rule, incomplete, not sufficiently comprehensive to do justice to the claimants; that very little thought was given to pathological sequence; that either great ignorance was displayed, or inexcusable carelessness manifested as to pathological results. It was then the rule, and not the exception, to accept as a pathological result anything that a claimant might allege, however contrary it might be to the best authorities. Furthermore, boards would complacently declare that because they could not account in any other way for the presence of a certain disability, it was of necessity due to the pensioned cause, notwithstanding there could be no possible connection between them.

A sailor alleged that he contracted "lead poisoning from eating fish that sucked the copper bottoms of ships," and a board of surgeons accepted such a relation.

One board reports that the claimant has "neuritis anterior branches of sciatic nerve from frosted feet," while another says: "Claimant complains of frequent urination, a condition caused by the excessive use of tobacco."

It is the experience of the medical examiners and reviewers of the bureau that too many boards have very little appreciation of their duties, and very little regard for the interests of the claimant or the government. It is too much a question with them of the number of examinations they make and the amount of fees that may be coming to them at the end of the quarter. The whole system of employment of the members of the boards of examining surgeons is faulty in this: that it fails to guarantee the best talent for these places. And so long as influence alone shall determine who shall be appointed, the bureau will fail to secure the best results.

It too frequently occurs that one is recommended and receives appointment on the ground that he stands well in his community as a general practitioner, but who by reason of lack of education is totally unfitted for the position of examining surgeon; who is not able to construct the simplest sentences correctly, misspells the simplest words, and shows in the description of disabilities an utter lack of professional knowledge, even confounding "mensuration" with "menstruation," "Eustachian" tubes with "Fallopian" tubes, etc.

A certificate was returned to an examining surgeon in North Carolina with directions to report the results of mensuration, palpation, auscultation and percussion of the chest. He returned it with the reply: "I did not know a man mensurated. I never heard of it before and I know no way they can."

A Tennessee examining surgeon declares: "I find him suffering with chronic rheumatism or lumbago of his hips and back, also with some heart trouble as *endometritis*."

A Kansas board reports: "Urine reaction neutral, *ophthalmoscope* reveals mucus and epithelial scales."

A Pennsylvania board reports that "By closing the mouth and nares and blowing, air passes through the *Fallopian* tubes and ears, and can be heard at a distance of twelve feet." Another Pennsylvania board reports "that his stools are thin and triangular in shape."

A Massachusetts board reports the soldier "to be suffering from a severe wound in the *right arm* of a long duration. It has the appearance of a bulliet wound entering at the one extremity of the tendo-Achilles and making its exit at the plantaries, just above the elbow, thus producing a partial blood poisoning and necropsy of the bone."

One of the New Jersey boards says: "There is evidence of *astigmatism*, the optic disc is oval and the vascular supply being all perpendicular."

I note these, but a few of the hundreds, to give you an idea of the difficulties that are encountered in the adjudication of claims—the ignorance, the carelessness and the indifference displayed by many examining surgeons.

It is to the credit of many boards that they take an honest pride in their work and are furnishing satisfactory reports. Their certificates show intelligence, care and thoroughness in the examinations, and an honest endeavor to report the actual condition of those examined. Too many others, however, apparently regard their duties and obligations in the lightest imaginable manner, their certificates bearing evidence of work done in a slipshod, haphazard manner. This faulty work is most frequently apparent in the inaccurate descriptions of disabilities and in the use of practically the same phraseology in describing necessarily varying forms of the same disability in different individuals. For instance, one board in twenty consecutive examinations found valvular disease of the heart and described it in practically the same words in each case. Test examinations failed to confirm these reports.

Many boards apparently have no conception or appreciation of the importance of their duties, and quiet their consciences by submitting machine-made reports that are in the highest degree unreliable and unsatisfactory. One board regarded its duties as fully performed when the secretary, having examined the claimants at his office, merely conferred over a telephone with the other members, after which all of them certified that they were present and personally participated in the examination.

Examining surgeons in their zeal for their patrons constitute themselves, in many instances, the agents of the claimants. I might instance the case of one who during a stated time participated in three examinations of a claimant, in each examination certifying to the fact contained in the certificate showing that the claimant was not disqualified for earning his support by manual labor, and yet, during the same period, filed five *sworn* statements testifying that the claimant was totally disabled for the performance of manual labor.

Both in personal interviews and correspondence, examining surgeons have declared their fears that by recommending just and equitable ratings they would excite the enmity of the claimant and his friends.

I invite attention to the conditions under the present system, to give you some idea of the difficulties with which we have to contend. It is, after all, not wholly the fault of the examining surgeons, but is, in a large measure, due to the system under which they are employed and assigned to work.

Under the practice which obtains, a board of surgeons is, after appointment, immediately assigned to work and proceeds to examine claimants—without previous instruction and without the remotest idea of what is required and absolutely necessary for the intelligent adjudication of claims by the bureau, and as a result, certificate after certificate must be returned for amendment, for a full and clear description of disabilities, and even then many claims must be medically adjudicated on imperfect and unsatisfactory reports of examining surgeons.

No business man could be induced to place a man in an important, responsible position with no prior experience or instruction in a single part of the work which he is called upon to perform, yet these examining surgeons, without experience, instructions or advice, are appointed and assigned to the work of furnishing to the government the data upon which nearly $140,000,000 are disbursed annually.

It has been the endeavor of commissioners of pensions to bring about a change in the system. I have, year after year, recommended, and the commissioner has urged that these surgeons should undergo an examination to test their scholastic and professional fitness for the place, and that after having shown their fitness, they should undergo a course of instruction in the examination of a claimant, and in the construction of certificates. So far, no disposition has been shown on the part of Congress to make any change, and we must get the best possible results under the existing system.

My excuse for dwelling at such length on this phase of the examining surgeon's duties is the fact that the results of examinations are so unsatisfactory; that many boards fail to understand the importance of their duties, and do not appreciate that the government must be considered as well as the claimant; that the government pays nearly a $1,000,000 a year to the examining surgeons of the Pension Bureau; and that their reports—these certificates—are made the basis for the disbursement of $140,000,000 of the government's money every year.

Many examining surgeons have expressed their fear that in doing their duty fearlessly they will lose the good-will of soldiers residing within the limits of their respective practices. They forget that the medical referee, and through him, the commissioner of pensions must assume the responsibility of disposing of the claims upon their individual merits. They fail to realise the magnitude of the work; they do not appreciate the fact that the whole output of the annual appropriation for pensions is made chiefly on the reports they furnish and the recommendations they make to the bureau.

Let me cite the following copy of a certificate made in a claim for ventral hernia by a board in a city of Illinois, as late as October, 1898, to give you an appreciation of the difficulties with which the bureau has to contend under the present system, and the necessity of rigid scrutiny of the reports of examining surgeons, and the absolute necessity for reörganising some boards:

We find that when the right side of the abdomen is not supported, the contents drop down into the iliac region. The attachments of the diaphragm have torn loose, the contents of the abdomen have thus fallen down, it is impossible for him to have an action of the bowels, so that it is necessary to raise them up. It causes him great pain when they are down, sometimes rigor. In order to hold them up he has to ware a wide, hard band around the body which has to be stuffed with pleggets of cloth, where it is not perfectly tight. This band has to be as tight as he can possibly make it. Mucous membrane of rectum relaxed. Vessels engorged, one tumor on each side, one inch in diameter.

The medical referee and the more than sixty medical men under him, dispose, on an average, of about 800 claims a day, and necessarily, a large number of these must be rejected, for the reason that many claimants are receiving the maximum rate that can be granted them under the laws, and many others are shown to have no ratable disability.

This responsibility these officials must assume, whatever unfriendly criticism may be passed upon them. Should not the examining surgeons assume theirs as well? The acceptance of the position and the oath of office they take before entering upon their duties, carry with them the promise that their reports will be unbiased and impartial and those who have not the courage to make such reports should resign.

Instead of this, however, too many surgeons, after writing reports, fearing that the bureau will take these at their actual worth, recommend ratings that are not warranted by their descriptions. When the claimants charge that the failure of the bureau to grant a rate as large as they hoped for is due to the report of these surgeons, they say: "We gave you a good rating—it is the commissioner of pensions and those under him who have rejected your claim." You can readily understand how this embarrasses the bureau.

I have said that one prominent cause for the receipt of so many unsatisfactory certificates is the fact that examining surgeons receive no instructions prior to entering upon their duties. No one appreciates this more than the commissioner of pensions and his medical referee. These officials have not the means of correcting this. They must execute the laws, and must do it with the means that Congress gives them.

It was believed, however, that much could be done to assist the examining surgeons by furnishing them with full instructions for their guidance. With this in view, the book of instructions was carefully revised. In its present form it has received the commendation of the Surgeon-general of the Army, and is used in the school of instructions for newly-appointed medical officers of the army. It has been supplemented by special instructions and by a carefully constructed letter indicating the points specially required of examining surgeons. It is believed that any fairly intelligent examining surgeon, if he familiarises himself with these, and has an honest desire to do his whole duty, cannot fail to give satisfactory reports.

There was a time when all that was required of examining surgeons was to give a description of the disabilities for which pension has been granted, or for which pension was claimed. As time elapsed, it was found that this was not sufficient. After a lapse of a quarter of a century since the close of the war of the rebellion, claims began to be filed for disabilities that were alleged to have been incurred during the war and in the service. No record of the existence of such disabilities in service was found in the War Department, and with so many opportunities for contracting them since, it became necessary, so as to protect the government against false claims, to secure, as far as possible, a trustworthy record of the physical condition of each claimant at the first opportunity.

The need of a thorough examination of every disability found, whether alleged or not, became imperative on the passage of the Act of June 27, 1890. This was made necessary under the requirement of the act that each claimant's capacity for earning a support by manual labor must be considered in the adjudication of the claim.

Section 2 of the Act provides "That all persons who served ninety days or more in the military or naval service of the United States during the late war of the rebellion, and who have been honorably discharged therefrom, and who are now or may hereafter be suffering from any mental or physical disability or disabilities of a permanent character, not the result of their own vicious habits, which so incapacitates them from the performance of manual labor, as to render them unable to earn a support, shall, upon making due proof of the fact, according to such rules and regulations as the Secretary of the Interior may provide, be placed upon the list of invalid pensioners of the United States and be entitled to receive a pension not exceeding $12 per month and not less than $6 per month, proportioned to the degree of inability to earn a support; and in determining such inability, each and every infirmity shall be duly considered, and the aggregate of the disabilities shown be rated.

The book of instructions, edition of 1900, aimed to inform examining surgeons of the differences in the requirements under the general laws, and the Act of June 27, 1890. On its receipt by examining surgeons, the bureau was flooded with letters asking for explanations and interpretations. So little attention was paid to these instructions; such a great degree of inharmony existed between the conditions actually described and the rates recommended; such a large number of certificates were furnished showing indifference and machine methods in the construction; so large a per cent. had to be returned for better descriptions or to have the ratings and descriptions harmonise—in fact, such an utter lack of compliance with the requirements of the instructions was shown, that, on June 30, 1900, a special circular was formulated and mailed to each examining surgeon in the United States.

This circular, as you remember, quoted the second section of the Act of June 27, 1890, as amended by the Act of May 9, 1900, and this was followed by specific instructions, to-wit:

The basis of ratings under this section is inability to earn a support by manual labor due to any mental or physical disability or disabilities of a permanent character not the result of the claimant's own vicious habits. There are *no schedule rates for incapacity resulting from any separate or combined disabilities.* If a pension is allowed under this section, it will be either for $6, $8, $10, or $12 per month, $6 being the minimum and $12 the maximum.

The Act of April 4, 1900, requires that the report of examining surgeons shall specifically state the rating to which, in their judgment, the claimant is entitled. This will be determined by giving due consideration to each and every infirmity, and rating the degree or extent to which all of these infirmities, whether minor or serious, when considered together, render the claimant unable to earn a support by manual labor. The result will be stated as \$6, \$8, \$10 or \$12 per month, and not as 6/18, 8/18, 10/18, etc., nor should there be a separate rating of separate disabilities as heretofore.

When in the judgment of the examining surgeons the aggregate of the disabilities disables the claimant in a degree materially affecting his capacity for earning a support by manual labor, the minimum rate of \$6 per month will be stated, and when the claimant is wholly unable to earn a support, or nearly so, by reason of his infirmities, the maximum rate of \$12 per month will be stated. For intermediate degree of disability, the rate of \$8 or \$10 per month will be stated according to the degree of disability.

Bear in mind that the maximum rate of pension under this section is \$12 per month, and that even if the claimant should be so disabled as to require the regular aid and attendance of another person, a higher rate cannot be allowed and should not be recommended.

The age of the claimant is an important factor in fixing the rate. A claimant who has reached the age of seventy-five years is allowed the maximum rate for senility alone, even where there are no special pensionable disabilities. A claimant who has attained the age of sixty-five years is allowed at least the minimum rate, unless he appears to have unusual vigor and ability for the performance of manual labor in one of that age. The effect of partial senility should be considered with other infirmities, where there are such, and the aggregate incapacity rated.

The certificate must state whether the several infirmities are permanent, and whether they are the result of the claimant's own vicious habits. Only those which are of a permanent character, and not the result of the claimant's own vicious habits can be considered in fixing the rate.

The certificate must give a full and accurate description of each and every disability in accordance with general instructions issued February 16, 1900.

It is desirable for the proper adjudication of the claim that each report of examination shall contain an exact pen picture of the condition of the claimant and an accurate and painstaking diagnosis of his infirmities in such detail, as to medical terms and descriptions, as will enable the medical officers of the bureau to determine accurately the condition of the claimant.

It was hoped that by a careful study of these special instructions, each board of examining surgeons would be able to furnish satisfactory reports. It is evident that it was carelessly read by many, or not read at all, because the bureau receives almost daily letters similar to the following:

This Pension Examining Board has received several communications from you recently instructing us in our duties as examiners, but nevertheless, we have to admit that we feel that our understanding of your requirements in our recommendations of ratings under the act of June 27, 1890, are not clear and definite enough, which vagueness we attribute largely to your qualifying phrases, to your definite statements.

By the book of instructions (Ed. 1900) specific rates are named in the tables and among these are five rates below the minimum rate under act of June 27, 1890, and in connection with those rates the first two lines particularly of paragraph 127, Book of Instructions, have led us to giving weight and consideration to such minor disabilities in our aggregate for a rate under the Act of June 27, 1890.

Please advise us whether we shall give weight and consideration to such minor disabilities when two or more of them would aggregate $6, the minimum rate under Act of June 27, 1890.

It must be evident that examining surgeons who will write such a letter can have no conception of the intent of the law or of their duties. What intelligent surgeon, or layman even, would regard an ordinary nasal catarrh, so common in this latitude; the loss of a part or the whole of a finger; slight deafness of both ears, or total deafness of one ear; a few small non-inflamed piles; a slight bronchitis; a varicocele; a small hydrocele; creaking of the joints without limitation of motion; a liver extending a fraction of an inch beyond the accepted average limit; an uncorrected refractive error; a senile arc; a presbyopia; a small ventral hernia; the loss of some teeth; and many other minor disabilities, as disabling a man for earning a support by manual labor?

Every day a number of certificates are received wherein boards declare that the aggregate of disabilities for earning a support by manual labor is due to one or more of these trifling ailments, and recommend a rating therefor of from $6 to $10.

A large per cent. of boards exercise no judgment as to what constitutes a factor in the causation of an inability to earn a support by manual labor. For example, a board in Oregon, under date of April 2, 1902, concludes a certificate with this statement:

We find that the aggregate permanent disability for earning a support by manual labor is due to his disposition and Christian belief, not due to vicious habits, and warrants a rate of $6.

I can account for this only in two ways. First, that no thought is given to the matter, and second, that the members of these boards did not experience the delight of earning their bread by the sweat of their brows, previous to entering upon their professional career. Surely, if they had the experience in this direction that some of us have had, they could appreciate the fact that even a number of more serious disabilities than these would be no appreciable handicap in the workingman's race for subsistence.

It seems exceedingly difficult for many boards to differentiate between the principle of ratings under the general law and the Act of June 27, 1890.

It appears to me that the whole difference is expressed in this, that under the general law it is a question of a *disability for the performance of manual labor*, for which the ratings range from $2 per month for a very slight disability, to $30 per month for total disability for manual labor; while under the act of June 27, 1890, it is a question of a disability for *earning a support* by manual labor, for which the lowest rating is fixed at $6 per month, and the highest at $12 per month. The disability warranting the maximum of $12 per month under the Act of June 27, 1890, must be practically as great as that which entitles to $30 per month under the general law. Surely it should not be difficult to distinguish the difference in the method of rating under these laws.

Neither the commissioner of pensions, the medical referee, nor the examining surgeons, have any right to let sentimental feelings control the performance of their duties. They must comply with the laws as Congress gives them, and as the secretary of the interior construes them. It is not a question of what you or I would like to do, but a question of doing our whole duty under the laws. It is not a question of what we in our sympathy may believe should be done, but of what the law requires us to do.

Many boards of examining surgeons continued to exhibit great indifference in the manner in which certificates were constructed, gross ignorance of their duties or incompetency to make a thorough systematic physical examination, or persisted in rating on subjective symptoms—the statements of the claimant or the representations of the attending physician, especially if he happened to be a member of the board—notwithstanding instructions to the contrary.

The special letter of instructions of January 3, 1901, was mailed to each examining surgeon, inviting attention to the defects in the examinations and the violation of the instructions.

Each was advised that the commissioner had been compelled to dismiss a number of boards, and that a continuance of such conduct would result in dispensing with the services of those offending.

Boards were also advised by the commissioner that he would "insist that examining surgeons, in rendering opinions as to pathological results, shall be guided by the best and most recent authorities on pathology and not, as is too frequently the case, accept conditions as pathological sequences when neither medical authority nor common sense warrants such acceptance."

Boards were further advised that "An examining surgeon who is not fully informed in the best and most recent authorities on pathology is of very little use to the bureau; on the contrary he is an embarrassment, a hindrance to the proper adjudication of claims." He declared it to be his determination "to secure for the government and the claimants the best talent obtainable, . . . . and to replace all examining surgons who are found to be incompetent or indifferent, with those who are competent and who are also willing to give their services to the performance of their official duties."

Since then a more careful scrutiny of the certificates has been made, with the result that the services of quite a number of examining surgeons have been dispensed with and other surgeons have been appointed in their places. Now, much better certificates are received, although quite a number of boards do not yet measure up to the standard desired.

Let me invite your attention to some of the more important errors that certificates of examination contain.

One of the most unsuccessful efforts has been that of making examining surgeons appreciate that pensions can only be predicated on objective conditions—on actual pathological changes. It is a very common occurrence for a board to make a statement similar to the following: "Soldier contracted diarrhea in 1864 and has suffered with it since. He has from ten to twenty evacuations daily. Sometimes he passes blood; he is worse in summer than in winter, and we find his disability total, entitling him to $12 per month." Not a single rational symptom is given; the certificate could as well have been written by a layman as by a physician; and yet the claimant was ordered for examination by a physician, for the reason that only a physician could give a diagnosis and sustain the statement of it by giving the rational and physical signs. Such a certificate is worthless for the reason that the surgeon knows absolutely nothing of the accuracy of the statement. Another form of statement, equally as worthless, is as follows: "Soldier was wounded at the battle of Gettysburg, in 1863, remained in hospital until November and was discharged. He has been unable to earn a support ever since." Again, certificates contain statements like the following: "This claimant contracted rheumatism at Petersburg, 1864; it affects his shoulders, back and hips. His shoulders are stiff, he can only raise arms to horizontal; he cannot stoop to reach the floor with his fingers; he cannot flex thighs, and is entitled to 17/18." The surgeons in stating the time and place of incurrence of the disabilities, certify to that of which they know nothing. It is frequently the case that claimants cannot prove the incurrence as they claim. Even if a surgeon did state it is of his own knowledge, the statement is of no value to the bureau, which must determine all such matters by reference to the records of the War Department.

The question in every case is *what does a careful examination show?* If a pathological condition exists, it should be so fully and clearly described that the medical officers of the bureau can understand the claimant's condition as fully and intelligently as did the surgeon who examined him. To do this a statement of limitation of motion of joints, based upon the claimant's voluntary movements, cannot, for obvious reasons, be accepted as conclusive. It must be based on the results of passive motion, and on this alone. Again, a statement that a claimant cannot stoop to touch the floor with his fingers, or reach it within more than a certain number of inches, furnishes no information. So much depends on the man's occupation, the size of his abdomen, whether he has practised athletic exercise, and, more particularly, his inclination, or rather his disinclination. Only the objective conditions—the pathological change in the physiological structures of the joints, and the muscles of the back, can be taken to determine the degree of disability.

Statements of few or many dejections due to diarrhea prove nothing. A clear, comprehensive pen picture of the conditions of all organs affected by this disease, including the general nutrition, must determine the pensionable degree of disability.

Boards too frequently are influenced by the plaintive story of suffering, made, as a rule, to influence their action, instead of by the actual condition as shown by a careful and thorough physical examination. They add the value of a rather insignificant disability to the value which their sympathies place on the claimant's plaintive story and rate, probably $24, or even $30, for a disability, their own description of which would warrant from $4 to $8 at the utmost.

If your patience is not exhausted, I will endeavor to point out specifically the most common defects in certificates of examination:

Failure to locate on the diagram all scars and losses of parts; to outline the form and extent of scars, ulcers and varicose veins, and the exact point of amputations.

A general disposition to recommend a rating somewhat in excess—say $2—of that which the claimant is receiving, notwithstanding that the disability is not progressive in character, such as permanently healed gunshot wounds or other old traumatic disabilities.

Duplicating and triplicating descriptions and rates for the same disability or diseased condition, as, for instance, when disease of stomach, chronic diarrhea and malarial poisoning are alleged, the pathological conditions due to which may properly be described and rated under either one of these designations.

Gunshot wounds and other injuries and scars are located on the diagram, and examiners content themselves by saying, "See diagram," without reporting the degree of deformity, loss of tissue, or impairment of motion and power resulting therefrom, although a rating indicating a serious disability is recommended.

The descriptions of rheumatism are often incomplete. One or more joints may be described as affected by swelling or stiffness, without giving comparative measurements and without reporting the degree of limitation shown on passive motion, the condition of other joints, muscles and tendons being left to be inferred by the medical officers of the Bureau.

In reports of the condition in disease of heart, the apex-beat is located so many inches below and to the right of the nipple, instead of by costal interspace and distance from *nipple line*. The conditions shown by auscultation are reported after exercise, instead of after claimant has been at rest. The question is frequently asked, "Why does the bureau refuse to accept the statement, "'the heart is normal?'" I answer, in the first place, it frequently happens that other certificates in the cases have reported organic valvular disease of the heart, and if the statement, "the heart is normal," were accepted as conclusive, the claimant's name would have to be dropped from the rolls. You can appreciate that it would be unusual and questionable to take such action on a statement of this character. Then, again, from such a statement alone it would be impossible to determine whether or not a careful examination of this organ was made.

The descriptions of the rectum are too brief—do not cover or embrace all the requirements of the instructions.

Failure on the part of boards to equip themselves with test lenses, and to give vision after the correction of existing refractive errors; failure to test vision in accordance with accepted methods. Pension cannot be granted for impaired vision which can be corrected by proper glasses.

The test for deafness is not made in accordance with instructions. While the distance at which the standard tones of voice *cannot* be heard is given, the distance at which they *can* be heard is not given. The latter is just as essential as the former. You will see the force of this statement when you are advised that slight deafness of one ear is "inability to hear ordinary conversation at six feet," and severe deafness of one ear, "inability to hear loud conversation at three feet." It oftens happens that a claimant refuses to admit that he can hear loud conversation at three feet, yet when off his guard, he hears ordinary conversation at one foot six inches, showing conclusively that he can hear ordinary conversation at some distance, and under the practice his deafness can only be rated as slight. Again, a claimant may claim "inability to hear loud conversation at three feet." The question is, does he hear it at any distance, for, under the rulings, so long as he can hear loud conversation at all, even with the lips to the ear, he can be rated for severe deafness only.

Failure to dispose of all disabilities alleged, and to paragraph properly; illegible penmanship; repetition of descriptions of a disability under different heads; separate ratings for each disability, instead of the aggregate rating in claims under the Act of June 27, 1890; rating deafness in eighteenths instead of thirteenths; using the statement "habits good," instead of the prescribed formula; only one witness, or two members as witnesses when claimant's signature is by mark; failure to state whether hernia is direct or indirect, or whether it passes the external ring; recommending excessive ratings not warranted by the pen picture—these are some of the more frequent defects in certificates.

While examining surgeons are prohibited from revealing the result of an examination to a claimant or other person, and from discussion or correspondence with a dissatisfied claimant, yet they are permitted to make affidavits or statements in pension claims of which they have professional or other knowledge, in the same manner as other physicians.

I have heretofore invited attention to the manner in which this privilege is abused, in the case of an examining surgeon who certified that the claimant had no pensionable disability, and then in a number of affidavits, no doubt furnished for "revenue only," swore that the claimant was seriously disabled.

In these cases, the certificate of examination is the bureau's sheet anchor, but in the claim of a widow under the general law, where her right to pension depends entirely on the death of her husband having been due to a disability of service origin or to a pathological result thereof, the only safety against affidavits that are made in the interest of a claimant, depends on the knowledge the medical officers of the bureau have of authoritative pathological sequences.

Only a few weeks ago a prominent physician of a noted summer resort of New Jersey furnished the death certificate to the state authorities that a pensioner's death was due to valvular disease of the heart. When the widow filed her claim, a sworn copy of this certificate was filed. The only disease for which legal approval could be made was neurasthenia, and consequently the claim had to be rejected on the ground that there was no pathological relation between neurasthenia and the disease of the heart from which the soldier died. The widow made an application for a re-opening of her claim, based on a long statement from the same physician. In this statement he swore that the soldier died from an acute attack of broncho-pneumonia, complicated with an acute attack of la grippe, accompanied with mitral insufficiency, aortic stenosis and general arteriosclerosis; and then argued that all these troubles were the direct results of neurasthenia. Re-opening was denied. He personally came to the bureau to argue the case with the medical referee. To the suggestion that he file a list of the authorities upon which his contention was based, he frankly admitted that he could find none. He further declared that had he known that his certificate would be used in a pension claim he would have made a different report. When asked why he did not give the death cause to the authorities as neurasthenia, he replied that the state medical authorities refused to accept it, knowing that the soldier died from other causes. He acknowledged that his certificate and subsequent statement contained the actual facts, yet he had the effrontery to declare that if he could withdraw them he would make the statement that the death was directly and immediately due to neurasthenia, so that the widow might get a pension. He was informed that such a statement would not be worth the paper it was written on, in any court, and then to his utter amazement he was informed that had he submitted such a sworn statement, making the so-called neurasthenia the death cause, the claim would necessarily have been rejected on the ground that syphilis could not be eliminated, for the reason that the soldier had a record of primary syphilis, a severe attack of syphilitic iritis, and, subsequently, all the manifestations of tertiary syphilis. This too, was a case of an affidavit "for revenue only." Cases of this character come under observation daily.

The following affidavit from an examining surgeon in Kansas, made in 1898, in another widow's claim, is to the point:

I made a thorough examination of him at that time, and made eight visits from that time until his death. When I made the visit in July I found that the spinal irritation had increased. His bronchitis had become so intense that his lungs was nearly overflowed with mucus and pus. He was expectorating profusely and in great quantity. His lungs was so full that it sounded like abscesses of his lung, but I am of the opinion that it was only an excess of mucus cutting air off from air cells which would give the lung symptoms. I am certain that the original cause of his death was rheumatism which caused paralysis of his back, said paralyzed condition allowed his spine to fall forward which caused pressure upon the spinal cord, and resulted in spinal irritation. The spinal irritation caused the nerves which supply the bronchial tubes and lungs to lose all sensation. Loosing said sensation, the mucus accumulated in excess in bronchial tubes and lung cells causing symptoms of abscesses which so diminish the airating surfaces he could not live. In such condition he died on August 22, 1897. While his immediate death was caused by overflow of mucus and pus in the bronchial tubes, the original cause of his death was rheumatism. The spinal irritation and excessive mucus and pus in bronchial tubes and lungs was results of rheumatism, causing paralysis of muscles of back, which resulted in spinal irritation and bronchitis and disease of lung tissue.

It is no surprise to the medical officers of the bureau to receive affidavits like this, but when a board of reputable physicians, in a certificate of examination dated February 5, 1902, makes the following statement: Applicant's muscular development is very poor, just why it should be so we are unable to state; the best we can describe it would be to say the muscles are slowly evaporating," it must be concluded that some examining surgeons regard their duties as a huge joke. Perhaps the board was influenced in accepting the theory of *evaporation* by the fact that the claimant's name was *Booze!*

I have said that the certificate of medical examination is the bureau's sheet anchor. It is the rule in the bureau to rely on these reports of examining surgeons and it is not usual to accept any evidence to controvert such reports. You can appreciate, therefore, the embarrassing position in which the medical officers of the bureau are placed when it is shown that examining surgeons have not been candid and honest in their reports, or when their reports show such a lack of general intelligence and professional knowledge as to make them unreliable.

About a year ago an affidavit was filed from a member of a board in Tennessee, declaring that a pensioner who was ordered for an examination under a claim for increase was confined to his house and unable to appear before the board. A member of the board was ordered to visit and examine this pensioner at his home. The surgeon's report showed that the claimant was so totally and permanently disabled as to require the regular and constant aid and attendance of another person. Some doubt was entertained as to the existence of this degree of disability and as to its permanency, because no such claim had been made by either the claimant or his attorney when filing the application for increase. Another member of the board was ordered to make a searching examination. His report confirmed the findings of the first examination, and the three members certified in the certificate of examination that each one was personally cognisant of the fact that the claimant required the regular and constant aid and attendance of another person, and was entitled to $72 per month. The increase was then allowed from the date of the first examination.

As soon as it became known in the neighborhood of the claimant that he was receiving such a high rate of pension, information was filed to the effect that he was not confined to his house at the time of the examination; that he walked to his place of business every day; that he not only personally superintended his business, but every few days drove to a neighboring town about six miles distant. On an investigation made by a special examiner, it was proven conclusively that this claimant had not been confined to his house, but attended to his duties daily. The claimant testified that he had not been confined to his house; that he had not required aid or attendance at any time, except for about two months four years prior to the examination, at which time he had an acute attack of rheumatism; and that he was in his usual health at the time of both examinations. In fact, it was shown, and the claimant admitted, that he was as active as men generally are who are seventy-two years old.

It is taken for granted that there must have been an inducement for making such a report. The board was suspended at once. After a suspension of one year, an application for reinstatement, urged by the member of Congress from that district and by resolution of the Grand Army post of the town, was denied by the present commissioner.

Three months ago another board reported that the apex-beat of the heart of a certain claimant was located under the xiphoid cartilage, the upper border of the heart under the sixth rib and the lower border under the ninth rib, with a mitral murmur. The certificate was returned inviting attention to the almost impossible location of the heart as reported. The board returned the certificate to the bureau insisting that the description was correct. A test examination showed that the apex-beat was at the sixth rib, that the heart was in the normal position, and that all its sounds were normal. The first certificate was rejected because it was unreliable and of no value to the bureau and the fee for examination was disallowed.

Conditions similar to these are encountered daily. Reports of boards lack candor, facts are suppressed, ignorance is displayed. You will, I am sure, appreciate the necessity for vigilance, and a careful scrutiny of the certificates of examination before applying them to the respective claims.

A board in Ohio, in thirty consecutive examinations of young men who served about three months during the war with Spain, and who were not outside of the United States, reported irritable heart in each case and declared that it was not due to vicious habits. This was so at variance with the experience of the bureau that a special investigation was held. This investigation elicited the information that where the pulse-rate was increased in frequency the heart was reported irritable. When the members of the board were asked to account for the accelerated pulse-rate, they gave it as their opinion that it was due to the excitement of the examination and to cigarette smoking. If this statement had been given in the reports of the board, an investigation would have been unnecessary and the board would have been advised that any disability due to the cigarette habit must be excluded in the consideration of a pension claim under the general law.

In concluding, I desire to express my appreciation of the honor and privilege of appearing before so many of my learned brothers.

It has been our aim for five years to improve the service of examining surgeons. We have but one purpose—that of giving to deserving pensioners all the pension they are entitled to, and to deny pensions to the undeserving. No one could possibly appreciate more than we, the necessity for a full and comprehensive pen picture of their disabilities in the certificates of examination, whereon to predict such pensions. We have aimed to improve the service of the examining surgeon so that claimants might receive what was due them.

The relations between the medical referee and the examining surgeons are necessarily, of a close and confidential nature. This official has always solicited from examining surgeons correspondence relating to any matter in which information was desired and he has always given a prompt reply.

If, perhaps, this paper has been too long, or not as interesting as you may have anticipated, the earnest desire for the very best work obtainable from you must be the apology, and if I have succeeded, at least in a measure, in impressing you with your personal responsibility, I am repaid.

If, perchance, the medical referee has found it necessary to return certificates to any of you; or you have been advised that more satisfactory reports must be furnished, or that a reorganisation of your board must be made, let me assure you that it has been done without malice or feeling, with no thought except for the best interest of those whose claims are at issue, and of the government, whose interest must also be considered.

I might quote you the familiar saying:

> "While the lamp holds out to burn
> The vilest sinner may return."

It is the policy of the bureau, as it is to its interest, to make as few changes of examining surgeons as possible. Its officials always protest against the displacement of experienced examiners who give satisfactory results; while, on the other hand, they insist that members of Congress recommend others in the place of those who fail to render satisfactory reports, or persist in violating the requirements of their instructions.

The progressive physician is one who is constantly looking out for that which will improve his mind and therefore improve his services to his patrons. The progressive examining surgeon is one who is on the alert for that which will assist him to make his services more valuable to the interests he represents. He, therefore, enjoys a conference with his fellow surgeons, and profits by an exchange of views. He furnishes the very best reports to the bureau. His certificates are models in every respect.

Your presence here today indicates that *you* are giving satisfactory service; that you have more than the usual interest in the performance of the duties incident to your responsible positions; that you are *progressive;* that you appreciate that, as in the great profession at large, there is benefit in occasionally meeting in a body and comparing methods, in discussing means to ends, in exchanging plans, in brightening your intellectual faculties by bringing them in contact with others. There should result, and, I have no doubt, there will result, a two-fold benefit. Each one present will be benefited, and the service of the bureau will be benefited. You will gain some new, some beneficial thought, you will look into the faces of many of your co-workers, you will appreciate better the interests you represent, you will carry home with you the remembrance of the acquaintances you have made, and their interest and enthusiasm in their work will be an inspiration to you for years to come. You will have a higher regard for your positions, and a better appreciation of the responsible duties of pension examining surgeons. You will have a more lively realisation that you have an honored and responsible part in the disbursement of $140,000,000 annually to deserving soldiers, their widows and orphans, and a deeper sense of the necessity for conscientious and comprehensive reports upon which such disbursements must be made, and by rendering which you will honor and dignify both yourselves and your office.

